# Peripheral Blood and Cerebrospinal Fluid Levels of YKL-40 in Alzheimer’s Disease: A Systematic Review and Meta-Analysis

**DOI:** 10.3390/brainsci13101364

**Published:** 2023-09-23

**Authors:** Yuchen Zhang, Jinzhou Tian, Jingnian Ni, Mingqing Wei, Ting Li, Jing Shi

**Affiliations:** Department of Neurology, Dongzhimen Hospital, Beijing University of Chinese Medicine, Beijing 100700, China; zhangyc@bucm.edu.cn (Y.Z.); jztian@hotmail.com (J.T.); jingnian_ni@hotmail.com (J.N.); mingqingwei001@126.com (M.W.); bjliting@yeah.net (T.L.)

**Keywords:** YKL-40, Alzheimer’s disease, blood, cerebrospinal fluid, biomarkers, meta-analysis

## Abstract

The pathogenesis associated with Alzheimer’s disease (AD) is particularly complicated, and early diagnosis and course monitoring of the disease are not ideal based on the available core biomarkers. As a biomarker closely related to neuroinflammation, YKL-40 provides a potential scalable approach in AD, but its association remains controversial and inconclusive with AD. We conducted this study to assess the utility of YKL-40 levels in peripheral blood and cerebrospinal fluid (CSF) of AD patients and healthy controls (HCs) by meta-analysis. We systematically searched and screened relevant trials for comparing YKL-40 levels between AD patients and HCs in PubMed, Embase, Cochrane, and Web of Science, with a search deadline of 14 March 2023 for each database. A total of 17 eligible and relevant studies involving 1811 subjects, including 949 AD patients and 862 HCs, were included. The results showed that YKL-40 levels in the peripheral blood of AD patients and HCs did not possess significant differences. Subgroup analysis showed YKL-40 significantly differed in plasma (SMD = 0.527, 95%CI: [0.302, 0.752]; *p* = 0.000), but did not in serum. In the case of comparison with HCs, YKL-40 was significantly higher in CSF of AD patients (SMD = 0.893, 95%CI: [0.665, 1.121]; *p* = 0.000). Besides that, when we performed a combined analysis of total YKL-40 in both peripheral blood and CSF, overall YKL-40 concentrations were also significantly increased among AD patients (SMD = 0.608, 95%CI: [0.272, 0.943]; *p* = 0.000). YKL-40 provides support and rationale for the neuroinflammatory pathogenesis of AD. The significance of CSF levels of YKL-40 for early screening of AD is definite. Plasma levels of YKL-40 also appear to assist in discriminating AD patients from HCs, which facilitates early screening and monitoring of the natural course of AD.

## 1. Introduction

Alzheimer’s disease (AD), particularly marked by an insidious onset, is a progressive neurodegenerative disease with a high prevalence and difficult diagnosis. Memory impairment, cognition disturbance, behavior abnormality, and communication disorders gradually manifest. There are currently no effective treatments to cure AD. It is of great significance to characterize and identify the early stages of AD owing to the long presymptomatic phase of AD.

At present, the diagnosis of AD mainly focuses on imaging examinations and the detection of cerebrospinal fluid (CSF) markers [1]. However, the expensive cost of positron emission tomography (Aβ-PET) scans and its inability to be widely available in clinical practice greatly hinder its feasibility. The sensitivity and specificity of biomarkers in CSF such as Aβ42, t-tau, and hyperphosphorylated tau (p-tau) concentrations have also raised doubts about their clinical significance with the continuous updates of modern research progress. Moreover, it is disheartening that numerous intervention treatment trials targeting such biomarkers have not achieved encouraging expected results and benefits. To improve the diagnosis and delay the process of AD, the development of biomarkers capable of detecting the early stages is of particular importance.

Research on biomarkers related to AD has significantly increased. Currently, Aβ42, t-tau, and p-tau remain core biomarkers for the early diagnosis of AD, despite their clinical significance decreasing with advances in modern research. Donovan A. McGrowder [2] evaluated new biomarkers for AD based on different pathological mechanisms such as neuronal injury, vascular dysregulation, synaptic dysfunction, and neuroinflammation. VILIP-1 may contribute to the identification of AD by mediating neuronal injury [3], but there was no significant difference in CSF levels of VILIP-1 between AD patients and Healthy Controls (HCs). Neurofilament Light (NFL) monitors disease progression by revealing neuronal degeneration in AD. However, the diagnostic potential of NFL is limited and lacks specificity compared to identified markers [4]. Plasma levels of neurogranin showed no significant differences between AD patients and HCs [5]. Synaptic proteins such as synaptotagmin and SNAP-25 are affected by AD, but their clinical significance requires further investigation [6]. The CSF levels of GAP-43 are elevated in the early stages of AD, but its diagnostic value is not well defined [7]. Iron homeostasis imbalance due to abnormal iron metabolism is one of the important factors in the pathomechanism of oxidative stress in AD [8], and there is no difference in CSF levels of ferritin in AD patients compared to HCs.

Moreover, studies have found correlations between AD and factors such as levels of Hcy (homocysteine) [9], ApoE ε4 (Apolipoprotein E ε4) [10], vitamin B6/B12 [11], S100B [12], folate, glucose metabolism (blood glucose, glycated hemoglobin, and serum insulin) [13], adiponectin, and thyroid-related hormones. However, due to various biological mechanisms and confounding factors like metabolic pathways, these biomarkers, while showing potential diagnostic value, require further research to determine if they can serve as independent risk factors for AD.

In the neuropathological study of AD inflammation, CSF levels of sTREM-2 were significantly higher in AD patients than HCs [14]. However, the diagnostic performance of sTREM-2 is not entirely stable, and plasma levels of sTREM-2 showed no significant differences. It was determined that the diagnostic value of GFAP is limited as it lacks substantial clinical utility in differentiating AD from other neurodegenerative disorders [15]. Elevated levels of MCP-1 have been associated with a variety of neurodegenerative and neuroinflammatory diseases, but MCP-1 was unable to distinguish AD patients from HCs [16]. Hs-CRP (high-sensitive C-reactive protein) may have diagnostic value for mild to moderate AD but cannot distinguish AD from HCs [17]. The pathologic process of AD is accompanied by a peripheral inflammatory response, and IL-6 (Interleukin-6) may be a useful biomarker [18], but its diagnostic utility for AD is inadequate. In addition, growing evidence suggests that the relevance of YKL-40 as a biomarker for AD is expanding [19], and CSF levels of YKL-40 may represent a novel and suitable biomarker with diagnostic utility for AD.

Excessive deposition of amyloid-β peptide (Aβ) and neurogenic fibrillary tangles (NFT) composed of hyperphosphorylated tau proteins as the main pathological features still fail to elucidate the pathogenesis of AD. Neuroinflammation is non-negligible in the pathogenesis and overall pathology of AD [20]. Modern research has found that neuroinflammation already occurs early in AD and acts as a critical component in the etiopathogenesis that may trigger disease progression [21]. Although biomarkers of neuroinflammation may be used to monitor the accuracy of early diagnosis of disease and feedback on outcomes after intervention, reliable and accurate biomarkers are still scarce [22]. Neuroinflammation is associated with chronic activation of astrocytes and microglia [23]. YKL-40, a type of carbohydrate-binding protein, is primarily localized in microglia, astrocytes, and peripheral macrophages, and plays an essential role in the induction and regulation of inflammation [24,25]. YKL-40 is considered to have important biological roles in inflammatory response and autoimmune diseases, reflecting the high inflammatory status and neuroinflammatory processes of different neurodegenerative dementia.

Mavroudis [19] demonstrated the effective benefits of CSF levels of YKL-40 in the definition of AD and analyzed in detail its ability to be applied in combination with the other core biomarkers to assess the prognostic effect of MCI and its correlation with AD disease progression. The research conclusion of Tizaoui [26] shows that YKL-40 is considered to be a specific marker in neuroinflammatory mechanisms, and that elevated YKL-40 can be detected during the course of AD. However, Hok-A-Hin’s [27] research data reported inconsistent results, suggesting that changes in YKL-40 obtained from extensive analysis using three different (semi) quantitative techniques may still not provide a valid and comprehensive understanding of YKL-40 alterations within the typical pathological brain tissue regions of AD.

Numerous studies aimed at assessing YKL-40 levels in AD have ended up with conflicting conclusions, which are evidently controversial. We carried out a comprehensive meta-analysis to explore differences in YKL-40 levels in peripheral blood and CSF between AD and healthy control subjects (HCs) and to investigate the role of YKL-40 in AD diagnosis.

## 2. Materials and Methods

### 2.1. Literature Search

This study was performed following the Preferred Reporting Items for Systematic Reviews and Meta-Analyses guidelines and was registered in the PROSPERO International Prospective Register of Systematic Reviews (https://www.crd.york.ac.uk/prospero/ (accessed on 9 April 2023)) (Number CRD42023412739).

We screened and collected relevant literature published up to 14 March 2023, after a systematic search by searching PubMed, Embase, Cochrane, and Web of Science. We used a combination of subject words and free words for retrieval. The terms and search strategy for the literature search was as follows: “(Alzheimer disease OR Alzheimer* OR dementia OR AD OR cogniti*) and (Chitinase-3-Like Protein 1 OR Chitinase 3 Like Protein 1 OR CHI3L1 OR YKL-40 OR YLK 40 OR Cartilage Glycoprotein 39 OR Glycoprotein 39 OR GP-39 OR GP 39 OR CGP-39 OR CGP 39)”. A manual search for all retrieved potentially relevant articles and reference lists was carried out to find additional available articles. The complete search strategy used is described in Supplemental Material.

### 2.2. Eligibility Criteria

#### 2.2.1. Inclusion Criteria

1. Human participants.
2. Samples from peripheral blood or CSF.
3. The study reports detailed groupings of AD and HC, along with the corresponding YKL-40 concentration values for each group.
4. The study describes the specific measurements of peripheral blood and CSF levels of YKL-40 samples.
5. Case-control studies or cross-sectional studies with complete and available data.
6. Written or published in English.

#### 2.2.2. Exclusion Criteria

1. Reviews, guidelines, letters, conference abstracts, commentaries, and case reports.
2. Medical history includes neurological, psychiatric, or other systemic disorders that may have an impact on cognitive function (e.g., depression, stroke, VaD, and mild cognitive impairment).
3. Lack of quantitative data on YKL-40 concentration and research with incomplete or unavailable data.
4. Failure to provide study data for mean and SD or SE or CI of YKL-40.

### 2.3. Data Extraction and Quality Assessment

Information to be derived from the literature obtained from the screening includes the lead authors of the paper, the year of publication, sample size, age, sex, ethnicity, AD criteria, measurement of YKL-40 method, YKL-40 level of mean and SD, SE or CI, and other characteristics including the score of the MMSE and acquisition of other available biomarkers. Two investigators independently screened the articles, cross checked and extracted the data and information, and if different opinions existed or consensus could not be reached, a third researcher was required to facilitate discussion and resolution. We assessed the quality of enrolled trials using the nine-star Newcastle–Ottawa Scale (NOS).

### 2.4. Statistical Analysis

All meta-analyses were conducted statistically using Stata (version 16.0) software. We performed statistical analysis by calculating the SMD and the corresponding 95%CI for continuous variable data as effect sizes to avoid statistical differences due to inconsistencies in the units of the means. For statistical heterogeneity, we used the *I*^2^ for testing and assessment. We decided to use a random effects model for the analysis, or else, when statistical heterogeneity was not significant (*p* > 0.05, *I*^2^ ≤ 50%), we chose to use a fixed effects model for the analysis. If only Standard Error (SE) data were available in the literature, the Standard Deviation (SD) was found by converting using the formula SD = √n × SE.

Possible sources of significant heterogeneity were assessed by subgroup analysis and further explored for factors with the greatest influence on high heterogeneity. The presence of publication bias was assessed by the observation of symmetry of the funnel plots, followed by Egger’s and Begg’s tests for further statistics and validation. Sensitivity analysis was used to examine if significant differences existed in individual studies or outliers that would markedly affect the robustness of overall study outcomes. Statistically significant differences were defined by a *p* value of <0.05, which applied to all test results in this study.

## 3. Results

### 3.1. Literature Search and Study Characteristics

**Figure 1:**
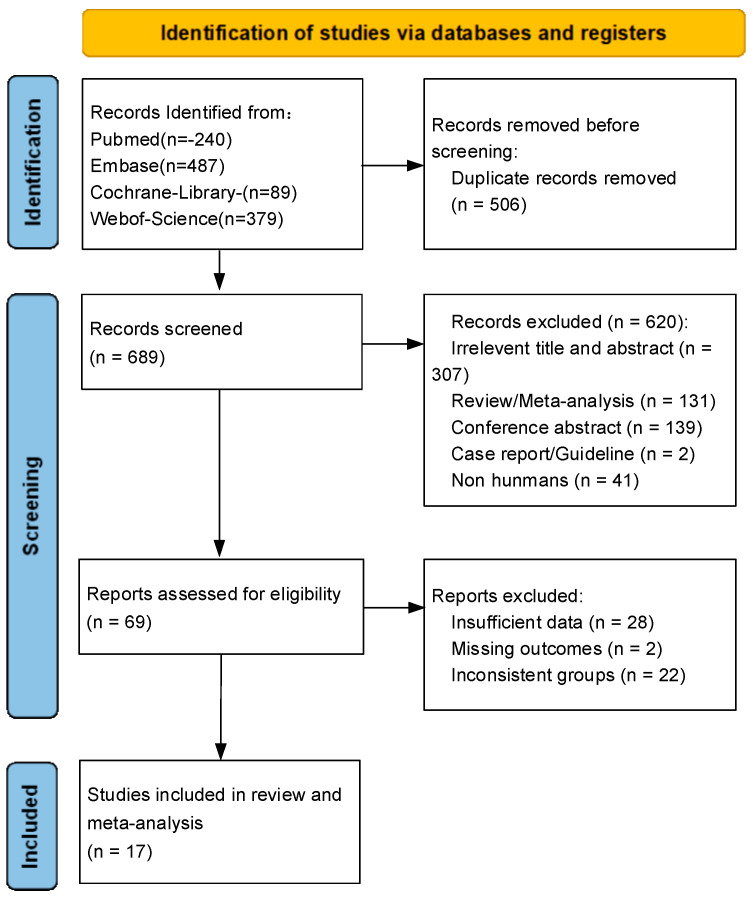
PRISMA flow diagram of research retrieval and screening process.

After the initial search, a total of 1195 eligible articles could be initially identified, including 240 in PubMed, 487 in EMBASE, 89 in Cochrane, and 379 in Web of Science. Of these, 506 duplicate articles were completely excluded in advance, after careful review of the titles and abstract contents of all the literature. After further detailed reading of the full-text content of 69 articles for screening, 28 articles were excluded due to insufficient data. Moreover, 2 articles were excluded due to missing outcomes, along with 22 articles that were excluded due to inconsistent grouping. In total, 17 articles were finally included. The search process of literature retrieval is presented in Figure 1.

A total of 17 studies were eventually included in this meta-analysis, which involved 1811 subjects, including 949 AD patients and 862 HCs. Among all included studies, 5 studies reported the levels of YKL-40 in peripheral blood (2 in plasma and 3 in serum, 1 in both serum and CSF) and 13 in CSF. Among all the subjects included in the study, 3 of the studies were Asian, while the 14 studies were Caucasian, and the average age range for all studies was 60–80 years. All included studies involved four diagnostic criteria for AD (eight NIA-AA, four NINCDS-ADRDA, two DSM-III-R, and one DSM-IIIR combined NINCDS-ADRDA), and one study provided detailed AD diagnostic measures but did not directly mention clear diagnostic criteria. The method for measuring YKL-40 levels in all studies was ELISA, except for one that used Meso Scale Discovery (MSD). In addition, some studies provided additional information about participants, including MMSE, CDR scores, and testing for what are currently considered core biomarkers such as Aβ, tau, and ApoE4. All specific basic information related to the included studies is shown in Table 1. The NOS quality assessment results revealed that most included articles were high-quality. The results of the specific NOS quality assessment are presented in Supplementary Material (Table S1).

### 3.2. Meta-Analysis of Peripheral Blood Levels of YKL-40 between AD and HCs

Five studies provided peripheral blood levels of YKL-40 in patients with AD and HCs, involving a total of 242 AD patients and 223 HCs (Table 1).

**Figure 2:**
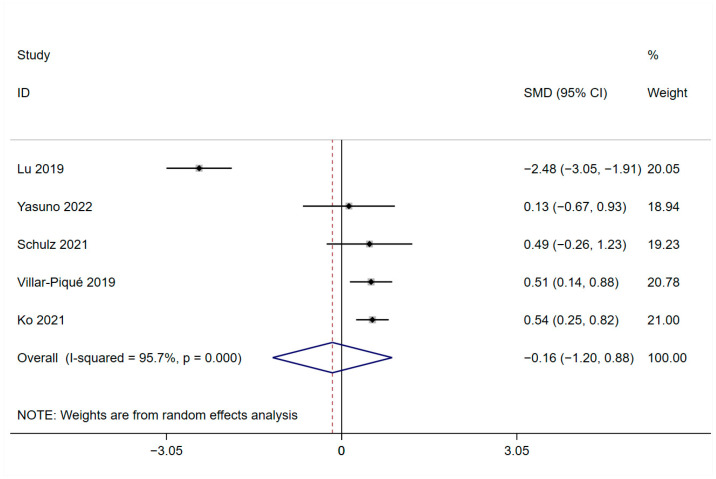
Forest plot of YKL-40 in peripheral blood of AD patients and HCs [20,28,29,30,31].

The results indicated that peripheral blood levels of YKL-40 did not differ significantly in AD patients and HCs (SMD = −0.16, 95%CI: [−1.20, 0.88]; *p* = 0.761; Figure 2). However, we found that high heterogeneity among the studies included in the analysis was present (*I*^2^ = 95.7%, *p* = 0.000). To further identify sources of high heterogeneity and the factors that most influence heterogeneity, we performed several subgroup analyses according to subject variation in YKL-40 sample source, race, AD diagnostic criteria, and mean age of AD patients (Table 2). The subgroup based on blood component samples (serum/plasma) analysis showed that the YKL-40 significantly differed between AD patients and healthy controls in plasma (SMD = 0.527, 95%CI: [0.302, 0.752]; *p* = 0.000), but not in the serum (SMD = −0.638, 95%CI: [−2.636, 1.361]; *p* = 0.532; Table 2, Figure S1). High heterogeneity still exists between studies in serum (*I*^2^ = 95.9%, *p* = 0.000), while none between studies in plasma (*I*^2^ = 0.0%, *p* = 0.920). In ethnicity subgroup analysis, peripheral blood levels of YKL-40 were significantly higher in Caucasian AD patients than healthy controls (SMD = 0.507, 95%CI: [0.176, 0.838]; *p* = 0.003). On the contrary, peripheral blood levels of YKL-40 did not significantly differ between studies of Asian AD patients and healthy controls (SMD = −0.605, 95%CI: [−2.598, 1.388]; *p* = 0.552). In addition, we found that in this subgroup analysis, a significantly high heterogeneity still existed between studies of Asian populations (*I*^2^ = 97.7%, *p* = 0.000), whereas no heterogeneity was observed between studies of Caucasian populations (*I*^2^ = 0.0%, *p* = 0.949; Table 2, Figure S2). The subgroup analysis according to the AD criteria revealed that YKL-40 levels were significantly higher in AD patients diagnosed by using NIA-AA than in HCs (SMD = 0.498, 95%CI: [0.281, 0.715]; *p* = 0.000; *I*^2^ = 0.0%, *p* = 0.637), but high heterogeneity remained between studies in NR group (SMD = −1.009, 95%CI: [−3.917, 1.899]; *p* = 0.497; *I*^2^ = 97.4%, *p* = 0.000; Table 2, Figure S3). In a subgroup analysis of patients with a mean age range of 60 to 69 years, it was noted that YKL-40 levels were statistically significantly higher in AD patients than in healthy controls (SMD = 0.527, 95%CI: [0.302, 0.752]; *p* = 0.000; *I*^2^ = 0.0%, *p* = 0.920). Studies of patients with an average age of 70 to 79 years have found significant heterogeneity in outcomes (SMD = −0.638, 95%CI: [−2.636, 1.361]; *p* = 0.532; *I*^2^ = 95.9%, *p* = 0.000; Table 2, Figure S4). Due to the limitations in the number of articles meeting the criteria in this part of the meta-analysis, we did not conduct further analysis.

### 3.3. Meta-Analysis of CSF Levels of YKL-40

**Figure 3:**
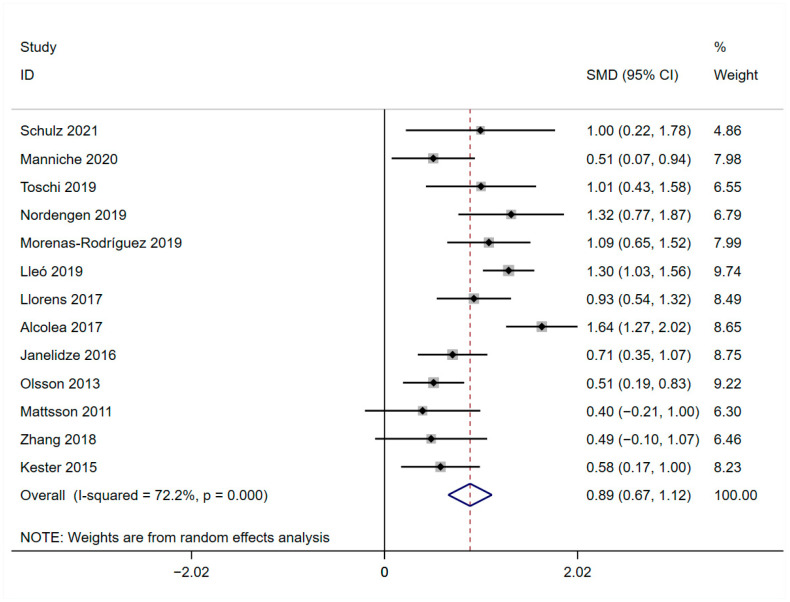
Forest plot of CSF levels of YKL-40 between AD and HCs [31,32,33,34,35,36,37,38,39,40,41,42,43].

Thirteen studies provided available data on the CSF levels of YKL-40 compared between AD patients and healthy controls, including 707 AD patients and 639 healthy controls (Table 1). The trend of elevated CSF levels of YKL-40 in AD patients compared to healthy controls was significant (SMD = 0.893, 95%CI: [0.665, 1.121]; *p* = 0.000; *I*^2^ = 72.2%, *p* = 0.000; Figure 3).

All studies carried out on CSF levels of YKL-40 were Caucasian in ethnicity. The subgroup analyses according to the AD criteria suggested that YKL-40 levels were higher in AD patients than in HCs in studies that used NIA-AA as the diagnostic criteria (SMD = 1.180, 95%CI: [0.825, 1.535]; *p* = 0.000; Table 3, Figure S5), but we could still observe high heterogeneity in the results of the analysis (*I*^2^ = 74.9%, *p* = 0.003; Table 3, Figure S5). When diagnostic criteria were used as the basis for subgroup analysis, we found that CSF levels of YKL-40 were significantly higher in AD patients than HCs in both studies with NINCDS-ADRDA (SMD = 0.763, 95%CI: [0.530, 0.996]; *p* = 0.000; Table 3, Figure S5) and DSM-III-R (SMD = 0.487, 95%CI: [0.205, 0.769]; *p* = 0.001; Table 3, Figure S5) as the diagnostic criteria, and heterogeneity between studies in both the NINCDS-ADRDA (*I*^2^ = 0.4%, *p* = 0.390; Table 3, Figure S5) criteria group and the DSM-III-R (*I*^2^ = 0.0%, *p* = 0.741; Table 3, Figure S5) criteria group did not exist. Moreover, one study did not explicitly report a proper name of the diagnostic criteria. In one study, DSM-IIIR combined with NINCDS-ADRDA was used as the diagnostic criteria for the included study population. The results of the age-based subgroup analysis suggested that YKL-40 levels were significantly elevated in the CSF of AD patients with a mean age between 60 and 69 years (SMD = 1.034, 95%CI: [0.689, 1.378]; *p* = 0.000; Table 3, Figure S6) and between 70 and 79 years (SMD = 0.823, 95%CI: [0.526, 1.121]; *p* = 0.000; Table 3, Figure S6) than healthy controls. However, high heterogeneity between studies remained, regardless of whether the participants included in the study were 60 to 69 years (*I*^2^ = 67.9%, *p* = 0.0818; Table 3, Figure S6) or 70 to 79 years (*I*^2^ = 73.1%, *p* = 0.1447; Table 3, Figure S6).

### 3.4. Meta-Analysis of Overall Levels of YKL-40

**Figure 4:**
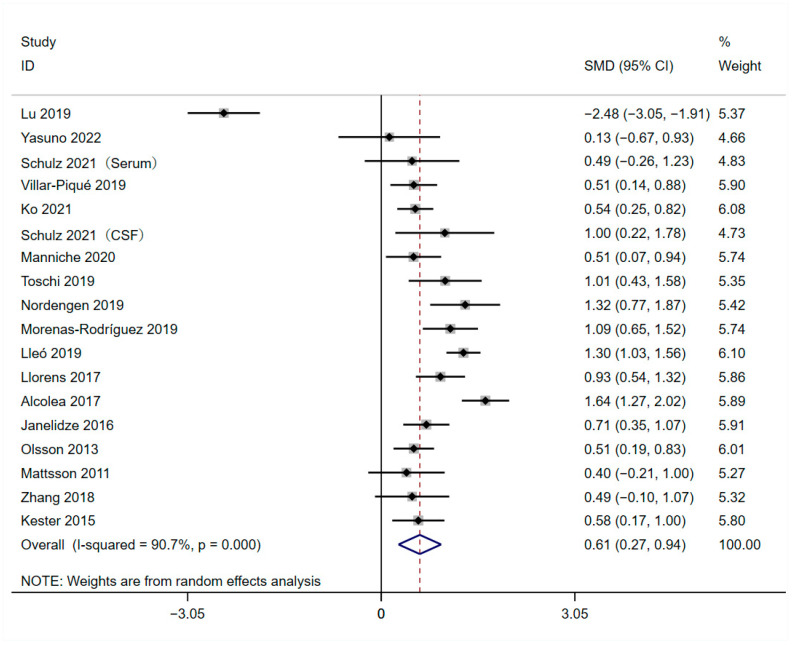
Forest plot of overall levels of YKL-40 between AD and HCs [20,28,29,30,31,32,33,34,35,36,37,38,39,40,41,42,43].

The 17 qualified studies included in this meta-analysis contained 949 patients with AD and 862 HCs (Table 1). The results of this meta-analysis indicated that overall levels of YKL-40 were found to be higher in AD patients than HCs (SMD = 0.608, 95%CI: [0.272, 0.943]; *p* = 0.000; Figure 4), and high heterogeneity was observed to exist across studies (*I*^2^ = 90.7%, *p* = 0.000; Figure 4). The significance of heterogeneity shown in subgroup analysis based on subject sample types of sources (Table 4, Figure S7), ethnicity background (Table 4, Figure S8), AD criteria (Table 4, Figure S9), and mean age range (Table 4, Figure S10) was inconsistent.

### 3.5. Results of Sensitivity Analysis and Publication Bias Analysis

**Figure 5:**
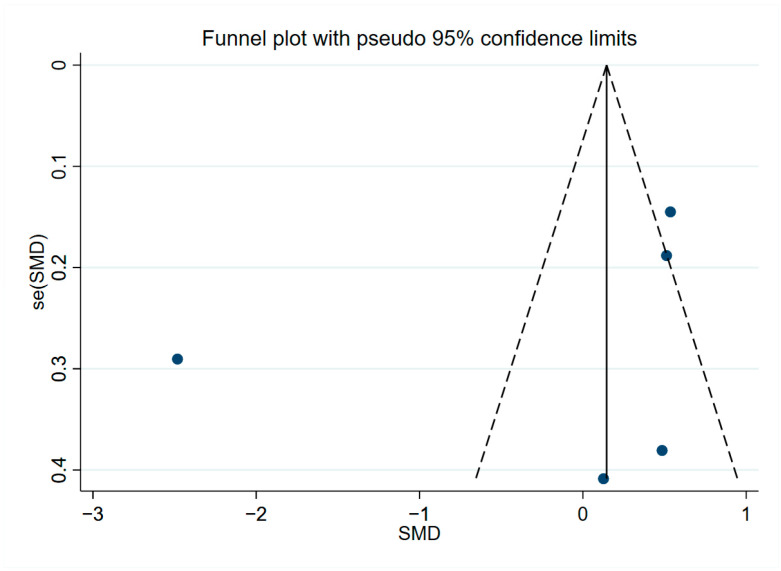
Funnel plot of peripheral blood levels of YKL-40.

**Figure 6:**
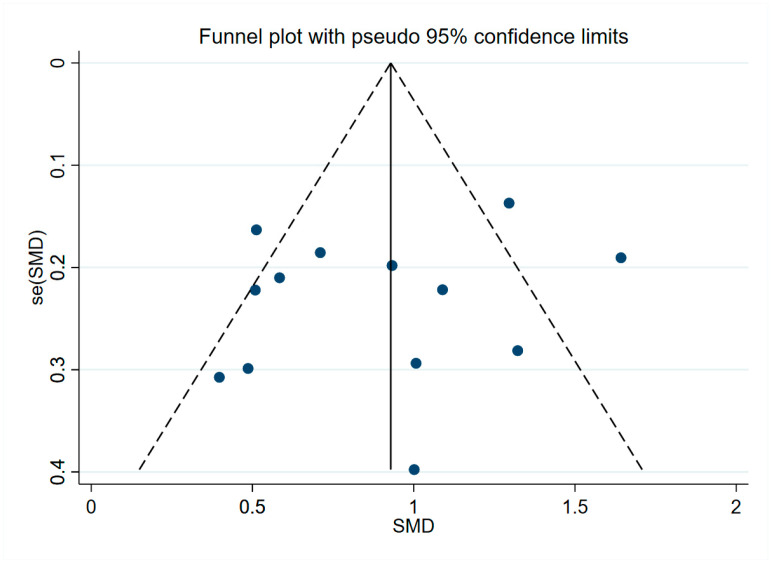
Funnel plot of CSF levels of YKL-40.

**Figure 7:**
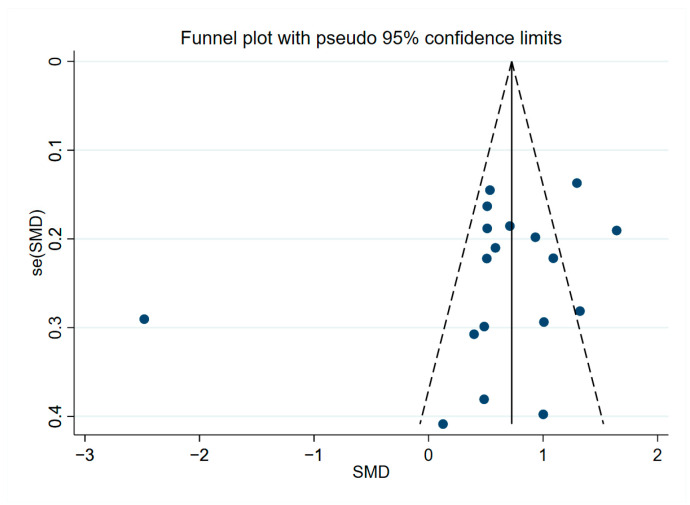
Funnel plot of overall levels of YKL-40.

The results of sensitivity analysis regarding YKL-40 in peripheral blood (Table S2), CSF (Table S4), and overall YKL-40 (Table S6) were shown to be robust. Analysis results of peripheral blood levels of YKL-40 by Begg’s test (*p* = 0.221, Table S3A) and Egger’s test (*p* = 0.468, Table S3B) suggested no publication bias present, and the results of the funnel plot assay were consistent with this (Figure 5). When we assessed CSF levels of YKL-40 with Begg’s test (*p* = 0.669, Table S5A) and Egger’s test (*p* = 0.455, Table S5B), no publication bias was observed, and the funnel plot assay demonstrated the same results (Figure 6). In addition, Begg’s test (*p* = 0.596, Table S7A) and Egger’s test (*p* = 0.239, Table S7B) were performed to assess the overall levels of YKL-40, and there was no evidence to support publication bias, and the same results could be observed for the funnel plot test (Figure 7).

### 3.6. Meta-Analysis of CSF Levels of Aβ42

**Figure 8:**
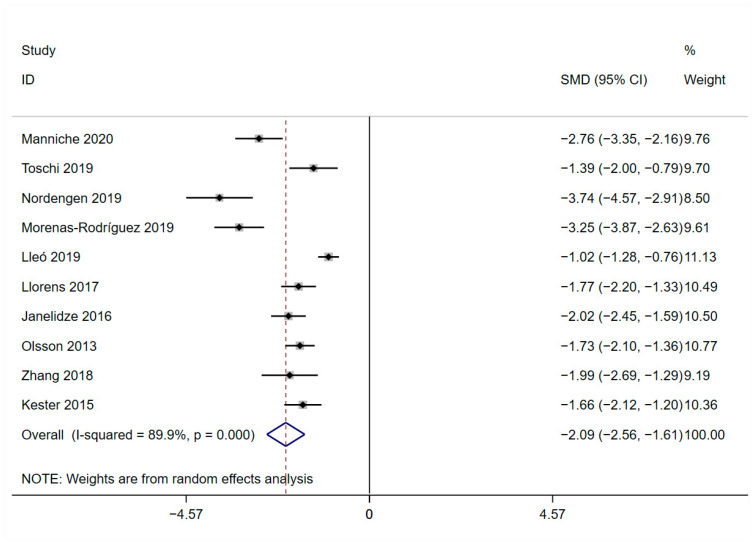
Funnel plot of CSF levels of Aβ42 [32,33,34,35,36,37,39,40,42,43].

Among the included studies, a total of 10 studies presented the expression of Aβ42, and all samples were from cerebrospinal fluid. The results showed that Aβ42 expression was significantly decreased in AD patients compared to HCs (SMD = −2.086, 95%CI: [−2.558, −1.614]; *p* = 0.000; *I*^2^ = 89.9%, *p* = 0.000, Figure 8).

It is clear that the “amyloid cascade hypothesis” alone cannot fully explain neuronal damage in AD. Amyloid cascade can be triggered by neuroinflammatory processes. Neuroinflammation plays a significant role in neurodegenerative diseases such as AD, and ongoing debates exist regarding its precise role [44]. We found that the results of the differences in Aβ42 levels between AD patients and HCs were consistent with the conclusions we drew about the differences in YKL-40. YKL-40 may play a crucial role in mitigating the development of AD by modulating neuroinflammatory responses and slowing down Aβ deposition.

## 4. Discussion

To our knowledge, this is the first meta-analysis to focus on peripheral blood levels of YKL-40 in AD patients and provides comprehensive insights into peripheral blood and CSF levels of YKL-40.

In modern research, neuroinflammation is considered to be a critical and prevalent pathophysiological mechanism in the disease process of AD. The conclusions of a systematic review that included 170 studies provide ample evidence to support the view that the pathogenesis of AD is accompanied by peripheral and CSF inflammatory responses [45]. YKL-40, also known as CHI3L1 (chitinase-3-like protein-1), is a secreted glycoprotein that is primarily expressed in reactive astrocytes upon inflammation conditions and considered to be a candidate as a neuroinflammatory biomarker of AD. It has been suggested that it is involved in neuroinflammation in AD as a pro-inflammatory molecule, contributing to the onset of the microglia activation process caused by amyloid accumulation, which leads to degenerative changes in the brain. Our systematic review elucidated the potentiality of YKL-40 in identifying AD patients and HCs. For this meta-analysis, our analysis results showed that plasma and CSF levels of YKL-40 were higher in AD patients than HCs, further demonstrating a significant correlation between elevated plasma and CSF levels of YKL-40 and AD, while the correlation between serum levels of YKL-40 and AD was not significant.

As can be seen, the focus of research on biomarkers of AD has gradually diverted toward blood biomarkers. Many studies are striving to identify available and effective AD blood biomarkers to avoid the invasive detection of CSF biomarkers and the expensive cost of PET scans. We performed subgroup analysis according to sample types of sources, ethnicity, diagnostic criteria of AD, and mean age range to analyze and evaluate YKL-40 levels in peripheral blood. We found no difference in serum levels of YKL-40 values between AD patients and HCs, whereas plasma levels of YKL-40 values differed significantly between the two groups, which may provide part of the evidence for considering plasma levels of YKL-40 as a potential peripheral biomarker for AD. The difference in plasma levels of YKL-40 corroborated a new alternative protocol that may help to identify neuroinflammatory processes in a less invasive way. However, in our analysis, the number of studies that ultimately met our inclusion criteria for peripheral blood levels of YKL-40 was not significant (three in serum and two in plasma). Moreover, we noticed that in the specific description of the diagnostic criteria for AD in Lu’s study [28], researchers did not use AD biomarkers or imaging detection measures. We suspect that this manipulation will have a certain impact on the final results for individual samples and even for the final analysis of the included studies.

Therefore, although the results of the current meta-analysis suggest the combined effect of YKL-40 in peripheral blood does not appear to be significantly different in identifying patients with AD and HCs, the finding is likely to be less reliable and additional studies are required to substantiate it in the future. In addition, it was found that peripheral blood levels of YKL-40 were higher in Caucasians with AD than HCs, but there were no differences between Asian groups. More research is required to determine whether confounding factors such as genetic factors, environmental factors, lifestyle habits, and economic conditions may be responsible for such differences.

CSF biomarkers are also another candidate for evaluating the activation of glial cells caused by neuroinflammation. In this meta-analysis of CSF levels of YKL-40, we originally hoped to identify and confirm the largest influencing factor of heterogeneity through subgroup analysis. The results suggest that inconsistent heterogeneity remains across studies based on AD criteria and mean age range subgroups. Therefore, we are currently unable to determine the source of heterogeneity. Despite some heterogeneity, our data demonstrate that CSF levels of YKL-40 are a sensitive biomarker in the neuroinflammatory process. A study [46,47] on neurodegenerative disease biomarkers in CSF suggests that YKL-40 is expanding its relevance as a pathological and physiological biomarker that not only distinguishes AD patients from HCs but also predicts the progression of dementia from preclinical stages to late stages [48]. This is consistent with our research results. In our meta-analysis, 13 studies reported the measurement of CSF levels of YKL-40, of which 11 simultaneously reported relevant test results involving one or more other CSF biomarkers including Aβ, tau, and APOE-ε4. This is also consistent with the viewpoint that the correlation between CSF levels of YKL-40 and core biomarkers of neurodegenerative diseases has been suggested in several studies [49]. Owing to the limited amount of available literature involved, we did not perform further and more specific analyses in this study. The results of autopsy pathology studies suggest that Aβ and tau pathology are related to astrocyte reactivity, and as a biomarker of astrocyte reactivity, CSF levels of YKL-40 may react differently in AD-related brain processes [50]. Another study [51] observed that CSF levels of YKL-40 mirrored astrocyte responses against tau tangle deposition in AD. Nevertheless, the evidence for the role of specific reactive astrocyte biomarkers related to Aβ and tau for disease progression in AD patients remains limited [52,53] in previous studies.

We combined the data of YKL-40 levels for analysis to verify whether the effects of overall analysis were consistent with the results of peripheral blood and CSF analysis alone. As evidenced by our findings, the overall levels of YKL-40 were significantly higher in AD patients than HCs. Meanwhile, we can see by the findings of this subgroup analysis that overall levels of YKL-40 exhibited significant differences in subgroups such as sample types, and AD criteria. This change might be related to direct CSF–blood exchange or other possible alternative mechanisms for regulating YKL-40 levels [54,55,56]. According to the results of modern studies, there is a definite association between the CSF levels of YKL-40 and plasma levels of YKL-40 that have been reported. One possible reason is that reactive astrocytes around blood vessels release brain-derived YKL-40 into the blood when cerebrovascular damage or blood–brain barrier damage occurs in AD. In this instance, the elevated peripheral blood levels of YKL-40 in AD patients reflect the pathological mechanism of neuroinflammation, but further research is necessary to shed light on the origin of peripheral blood levels of YKL-40 and to clarify if this indicates impairment to cerebral blood vessels.

It is worth mentioning that we found that YKL-40 levels seem to be associated with aging. The results of this study showed that YKL-40 levels in both peripheral blood and CSF were higher in AD patients aged 60 to 69 than in HCs, but this was not a significant difference for those aged 70 to 79 in peripheral blood. Also noteworthy, CSF levels of YKL-40 are influenced by age, which has implications for the early diagnosis and screening of AD. It further supports the use of YKL-40 to evaluate the progression of AD disease and monitor potential therapeutic interventions. Exactly as reported in a longitudinal study [57], there is an age-related increase in YKL-40 in AD individuals.

In the studies included in our analysis, three studies reported on ApoEε4 (Apolipoprotein Eε4), and only one study involved inflammatory cytokines IL-6 (Interleukin-6), IL-8 (Interleukin-8), and chemokine CCL-2. We attempted to explore the specific association of YKL-40 with the ApoE genotype and different cytokines as well as chemokines. Research has confirmed that APOE (Apolipoprotein E), the strongest genetic risk factor for AD, is synthesized and secreted by astrocytes and microglia, and the ApoEε4 allele plays a crucial role in neuroinflammation. Additionally, the degree of neuroinflammation in AD, characterized by the continuous interaction of chemokines and cytokines such as CCL-2, IL-6, IL-1β, and TNF-α, with Aβ, reflects the activity of neuroinflammation. Pro-inflammatory and anti-inflammatory factors bidirectionally regulate microglial polarization, and the balance affects the prognosis of AD. Prolonged pro-inflammatory activation can lead to increased secretion of inflammatory factors and toxic substances, exacerbating the inflammatory response. Anti-inflammatory action can maintain the stability of M2 microglial cells, increase Aβ internalization and degradation, and reduce the activity of inflammation. Chemokine CCL2, also known as monocyte chemoattractant protein (MCP-1), is a pro-inflammatory signaling protein produced by Aβ-associated microglia. Many studies consider CCL2 as an early inflammatory marker in AD. IL-6 is a crucial pro-inflammatory cytokine, while IL-8 is a multifaceted inflammatory cytokine, both of which are important indicators of systemic inflammation. IL-6 was found to be associated with future deposition of higher amyloid loads. It was shown that YKL-40 enhanced the upregulation of pro-inflammatory factors such as CCL2, IL-6, and IL-8, forming a positive feedback loop in the neuroinflammatory cascade, thereby accelerating the onset and progression of AD. The specific association of YKL-40 with the ApoE genotype and the involvement of different cytokines, as well as chemokines, in the neuroinflammatory mechanisms of AD is well established, but the limited available data in this study do not yet allow for deeper and more definitive conclusions.

This meta-analysis still had several limitations. First, since the sample size regarding peripheral blood in the studies ultimately included in this meta-analysis was limited, additional clinical studies containing larger sample sizes are necessary to validate the effect of peripheral blood levels of YKL-40 in AD. Secondly, in this meta-analysis, all comparative studies showed high inter-study heterogeneity, and we attempted to identify the factors that had the greatest impact on the observed high heterogeneity, but based on existing research, we were unable to conduct further analysis on other possible confounding factors such as genetic and environmental factors, lifestyle, and economic conditions. The exact mechanisms of neuroinflammation in AD pathology remain unclear, and the initial mechanisms of Aβ formation are not well understood. The imbalance between pro-inflammatory and anti-inflammatory cytokines may be one of the crucial mechanisms in AD development. However, most studies on cytokines and chemokines have only examined changes in their levels in AD and have not delved into the mechanisms by which these inflammatory factors affect microglial phenotypic transformation or Aβ-induced neuroinflammation. Therefore, future large-scale studies are needed to further elucidate the specific mechanisms of how inflammatory factors regulate microglial phenotypic transformation and whether improving the degree of neuroinflammation by increasing the positive effects of anti-inflammatory chemokines and reducing the negative effects of pro-inflammatory chemokines is a feasible approach for targeted AD treatment. The discriminative ability of YKL-40 obtained in this study seems to be limited. Owing to the unclear difference of YKL-40 among other neurodegenerative diseases, it still cannot be used as a specific biomarker for AD and reflects only the inflammatory process. Further evaluation is required to estimate the specific diagnostic accuracy of YKL-40 and the correlation with the risk of preclinical AD, MCI, PD, and other subtypes of dementia. Despite these limitations, our study may open the possibility of considering YKL-40 a dependable marker for providing clinical value in the early discrimination and evaluation of disease progression.

## 5. Conclusions

Our present study revealed that YKL-40 levels in plasma and CSF were substantially higher in AD patients than HCs, and serum levels of YKL-40 did not differ. Overall levels of YKL-40 were higher in AD patients. YKL-40 provides support and a rationale for the neuroinflammatory pathogenesis of AD. The significance of CSF levels of YKL-40 for early screening of AD is definite. Plasma levels of YKL-40 also appear to assist in discriminating AD patients from HCs, which facilitates early screening and monitoring of the natural course of AD. However, as evidence to support the diagnostic significance of YKL-40 in the framework of AD research, it remains inadequate. YKL-40 may reflect the inflammatory progression of the AD disease process. Considering that the pathological mechanisms of neuroinflammation in AD have significant commonalities with other neurodegenerative diseases, and although our current findings may indicate the prospect and potential of YKL-40 being one of the valid candidate biomarkers for AD, its applicability in the differential diagnostic contexts of AD and in monitoring the natural course of AD requires further exploration and additional studies with larger sample sizes to be clarified and validated.

## Figures and Tables

**Table 1:** Characteristics and data used for the studies included in this meta-analysis.

Study	Sample Source	Ethnicity	AD Criteria	Mean Age (y)	Method	Male/Female	Mean MMSE	Biomaterial
Lu 2019 [28]	Serum	China (A)	NR	AD = 79.77 ± 12.97, NC = 74.50 ± 6.45	ELISA	AD = 18/27, NC = 23/17	AD = 15.10 ± 5.78, NC = 29.06 ± 1.07	Fasting blood glucose, Triglyceride, Total cholesterol lipoprotein
Yasuno 2022 [20]	Serum	Japan (A)	NIA-AA	AD = 78.1 ± 3.9, NC = 78.9 ± 5.2	ELISA	AD = 7/8, NC = 4/6	AD = 20.7 ± 2.4, NC = 26.2 ± 2.2	ApoE4, BMI, PET
Villar-Piqué 2019 [29]	Plasma	Germany (C)	NIA-AA	AD = 69 ± 10, NC = 66 ± 5	ELISA	AD = 25/25, NC = 48/22	NR	NR
Ko 2021 [30]	Plasma	Korea (A)	NIA-AA	AD = 65.2 ± 9.9, NC = 64.7 ± 9.9	ELISA	AD = 51/70, NC = 36/47	AD = 17.3 ± 6.3, NC = 28.1 ± 1.9	CDR, Aβ42, t-tau, p-tau
Schulz 2021 [31]	Serum/CSF	Germany (C)	NR	AD = 74.27 ± 4.64, NC = 68.75 ± 6.38	ELISA	AD = 6/5, NC = 14/6	AD = 17.89 ± 7.72, NC = 28.20 ± 1.47	α-Synuclein, NfL, tTau, UCHL-1, GFAP, S100B, sTREM-2
Manniche 2020 [32]	CSF	Denmark (C)	NIA-AA	AD = 70.28 ± 8.0, NC = 64.52 ± 7.6	ELISA	AD = 30/27, NC = 19/14	AD = 23.44 ± 4.6, NC = 28.96 ± 1.3	NFL, NG, Aβ42, t-tau, p-tau
Toschi 2019 [33]	CSF	France/Germany/Sweden (C)	NINCDS-ADRDA	AD = 70.4 ± 7.7, NC = 60.7 ± 10.3	ELISA	AD = 18/19, NC = 7/13	AD = 21.7 ± 5.0, NC = 29.4 ± 0.8	p-tau181, t-tau, Aβ1-42, NFL
Nordengen 2019 [34]	CSF	Norway (C)	NIA-AA	AD = 67.6 ± 5.2, NC = 61.1 ± 9.2	MSD	AD = 14/13, NC = 17/19	AD = 19.0 ± 5.8, NC = 29.4 ± 0.7	ApoE4, Aβ42, t-tau, p-tau
Morenas-Rodríguez 2019 [35]	CSF	Spain (C)	NIA-AA	AD = 74.6 ± 5.6, NC = 67.4 ± 5.1	ELISA	AD = 19/31, NC = 19/25	AD = 22.5 ± 3.4, NC = 28.9 ± 1.2	Aβ1-42, t-tau, p-tau, sTREM2, PGRN
Lleó 2019 [36]	CSF	Spain (C)	NIA-AA	AD = 68.5 ± 8.5, NC = 58.2 ± 7.2	ELISA	AD = 63/47, NC = 68/86	AD = 22.6 ± 4.1, NC = 28.7 ± 1.2	APOE ε4, t-tau, p-tau, Aβ1-38, Aβ1-40, Aβ1-42, Aβ1-42/Aβ1-40, Aβ1-42/t-tau, NFL
Llorens 2017 [37]	CSF	Germany (C)	NINCDS-ADRDA	AD = 67 ± 11, NC = 70 ± 6	ELISA	AD = 22/43, NC = 23/27	NR	tau, p-tau, Aβ42, S100B, NSE, 14-3-3
Alcolea 2017 [38]	CSF	Spain (C)	NIA-AA	AD = 70.8 ± 7.8, NC = 60.2 ± 8.3	ELISA	AD = 28/44, NC = 31/45	AD = 21.6 ± 4.6, NC = 29.0 ± 1.1	NR
Janelidze 2016 [39]	CSF	Sweden (C)	DSM-III-R combine NINCDS-ADRDA	AD = 76.4 ± 7.4, NC = 75.3 ± 6.4	ELISA	AD = 24/50, NC = 16/37	AD = 19.4 ± 3.3, NC = 28.6 ± 1.8	Neurogranin, Aβ40, Aβ42, Tau
Olsson 2013 [40]	CSF	Sweden (C)	DSM-III-R	AD = 76.2 ± 7.4, NC = 74.7 ± 7.5	ELISA	AD = 34/62, NC = 17/48	AD = 19.0 ± 3.8, NC = 28.7 ± 1.6	Aβ1-42, t-tau, p-tau, sCD14
Mattsson 2011 [41]	CSF	Sweden (C)	DSM-III-R	AD = 74 ± 4, NC = 74 ± 5	ELISA	AD = 11/14, NC = 9/10	NR	CCL2, IL6, IL8
Zhang 2018 [42]	CSF	USA (C)	NINCDS/ADRDA	AD = 74.3 ± 6.8, NC = 76 ± 5.7	ELISA	AD = 7/11, NC = 19/13	AD = 24.2 ± 2.1, NC = 29.2 ± 1.1	Aβ42, t-tau, p-tau181, VILIP-1
Kester 2015 [43]	CSF	Netherlands (C)	NINCDS-ADRDA	AD = 65 ± 8.1, NC = 64 ± 12.2	ELISA	AD = 36/29, NC = 23/14	AD = 22 ± 5.6, NC = 28 ± 1.8	Aβ42, tau, p-tau181, VILIP-1

Abbreviations: AD, Alzheimer’s disease; HCs, healthy control subjects; CSF, cerebrospinal fluid; A, Asian; C, Caucasian; CDR, Clinical Dementia Rating; BMI, Body Mass Index; MMSE, Mini-Mental State Examination; NfL, neurofilament light chain; ApoE4, apolipoprotein E 4; PET, positron emission tomography; IL6, Interleukin-6; IL8, Interleukin-8; GFAP, glial fibrillary acidic protein; sCD14, soluble cluster of differentiation 14; S100B, S100 Calcium Binding Protein B; PGRN, Progranulin; NSE, neuron-specific enolase; sTREM2, Soluble triggering receptor expressed on myeloidcells 2; CCL2, C-C motif chemokine 2; VILIP-1, Visinin-Like Protein 1; ELISA, enzyme-linked immunosorbent assay; MSD, Meso Scale Discovery; NIA-AA, National Institute on Aging–Alzheimer’s Association; DSM-III-R, Diagnostic and Statistical Manual of Mental Disorders, 3rd Edition; NINCDS–ADRDA, National Institute of Neurological and Communicative Disorders and Stroke–Alzheimer’s Disease and Related Disorders Association; NR, Not reported.

**Table 2:** Subgroup analysis of peripheral blood levels of YKL-40.

Subgroups	*n* of Studies	SMD (95%CI)	*I* ^2^	*p*-Value
All studies	5			
Sample types Source				
Serum	3	−0.638 (−2.636, 1.361)	95.9%	0.532
Plasma	2	0.527 (0.302, 0.752)	0.0%	0.000
Ethnicity				
Asian	3	−0.605 (−2.598, 1.388)	97.7%	0.552
Caucasian	2	0.507 (0.176, 0.838)	0.0%	0.003
AD Criteria				
NIA-AA	3	0.498 (0.281, 0.715)	0.0%	0.000
NR	2	−1.009 (−3.917, 1.899)	97.4%	0.497
Mean Age Range				
70–79 y	3	−0.638 (−2.636, 1.361)	95.9%	0.532
60–69 y	2	0.527 (0.302, 0.752)	0.0%	0.000

Abbreviations: AD, Alzheimer’s disease; NIA-AA, National Institute on Aging–Alzheimer’s Association; SMD, Standardized Mean Difference; NR, Not reported.

**Table 3:** Subgroup analysis of CSF levels of YKL-40.

Subgroups	*n* of Studies	SMD (95%CI)	*I* ^2^	*p*-Value
All studies	13			
AD Criteria				
NIA-AA	5	1.180 (0.825, 1.535)	74.9%	0.000
NINCDS-ADRDA	4	0.763 (0.530, 0.996)	0.4%	0.000
DSM-III-R	2	0.487 (0.205, 0.769)	0.0%	0.001
NR	1	1.002 (0.222, 1.781)	-	0.012
DSM-IIIR combine NINCDS-ADRDA	1	0.710 (0.347, 1.074)	-	0.000
Mean Age Range				
70–79 y	9	0.823 (0.526, 1.121)	73.1%	0.000
60–69 y	4	1.034 (0.689, 1.378)	67.9%	0.000

Abbreviations: AD, Alzheimer’s disease; NIA-AA, National Institute on Aging–Alzheimer’s Association; DSM-III-R, Diagnostic and Statistical Manual of Mental Disorders, 3rd Edition; NINCDS-ADRDA, National Institute of Neurological and Communicative Disorders and Stroke–Alzheimer’s Disease and Related Disorders Association; SMD, Standardized Mean Difference; NR, Not reported.

**Table 4:** Subgroup analysis of overall levels of YKL-40.

Subgroups	*n* of Studies	SMD (95%CI)	*I* ^2^	*p*-Value
All studies	18			
Sample types of Sources				
Serum	3	−0.638 (−2.636, 1.361)	95.9%	0.532
Plasma	2	0.527 (0.302, 0.752)	0.0%	0.000
CSF	13	0.893 (0.665, 1.121)	72.2%	0.000
Ethnicity				
Asian	3	−0.605 (−2.598, 1.388)	97.7%	0.552
Caucasian	15	0.846 (0.634, 1.058)	71.3%	0.000
AD Criteria				
NIA-AA	8	0.908 (0.560, 1.256)	83.8%	0.000
NINCDS-ADRDA	4	0.763 (0.530, 0.996)	0.4%	0.000
DSM-III-R	2	0.487 (0.205, 0.769)	0.0%	0.001
NR	3	−0.344 (−2.635, 1.947)	96.9%	0.769
DSM-IIIR combine NINCDS-ADRDA	1	0.710 (0.347, 1.074)	-	0.000
Mean Age Range				
70–79 y	12	0.465 (−0.068, 0.998)	92.8%	0.087
60–69 y	6	0.852 (0.536, 1.169)	78.3%	0.000

Abbreviations: AD, Alzheimer’s disease; CSF, cerebrospinal fluid; NIA-AA, National Institute on Aging–Alzheimer’s Association; DSM-III-R, Diagnostic and Statistical Manual of Mental Disorders, 3rd Edition; NINCDS-ADRDA, National Institute of Neurological and Communicative Disorders and Stroke–Alzheimer’s Disease and Related Disorders Association; SMD, Standardized Mean Difference; NR, Not reported.

## Data Availability

No new data were created or analyzed in this study. Data sharing is not applicable to this article.

## Supplementary Materials

The following supporting information can be downloaded at: https://www.mdpi.com/article/10.3390/brainsci13101364/s1, Table S1: Newcastle–Ottawa Scale scores; Table S2: Sensitivity analysis of YKL-40 levels in peripheral blood; Table S3A: Publication bias of YKL-40 levels in peripheral blood (Begg’s Test); Table S3B: Publication bias of YKL-40 levels in peripheral blood (Egger’s test); Table S4: Sensitivity analysis of YKL-40 levels in CSF; Table S5A: Publication bias of YKL-40 levels in CSF (Begg’s Test); Table S5B: Publication bias of YKL-40 levels in CSF (Egger’s test); Table S6: Sensitivity analysis of overall levels of YKL-40; Table S7A: Publication bias of overall levels of YKL-40 (Begg’s Test); Table S7B: Publication bias of overall levels of YKL-40 (Egger’s test); Figure S1: Forest plot of subgroup analysis of YKL-40 by sample in peripheral blood of AD patients and HCs; Figure S2: Forest plot of subgroup analysis of YKL-40 by ethnicity in peripheral blood of AD patients and HCs; Figure S3: Forest plot of subgroup analysis of YKL-40 by AD criteria in peripheral blood of AD patients and HCs; Figure S4: Forest plot of subgroup analysis of YKL-40 by mean age in peripheral blood of AD patients and HCs; Figure S5: Forest plot of subgroup analysis of YKL-40 by AD criteria in CSF of AD patients and HCs; Figure S6: Forest plot of subgroup analysis of YKL-40 by mean age in CSF of AD patients and HCs; Figure S7: Forest plot of subgroup analysis of overall levels of YKL-40 by sample of AD patients and HCs; Figure S8: Forest plot of subgroup analysis of overall levels of YKL-40 by ethnicity of AD patients and HCs; Figure S9: Forest plot of subgroup analysis of overall levels of YKL-40 by AD criteria of AD patients and HCs; Figure S10: Forest plot of subgroup analysis of overall levels of YKL-40 by mean age of AD patients and HCs; Data sheet: All extracted raw data and basic information.
