# Supplementary material for: Peripheral Blood and Cerebrospinal Fluid Levels of YKL-40 in Alzheimer’s Disease: A Systematic Review and Meta-Analysis
Source: Brain Sci. 2023 Sep 23;13(10):1364. doi: 10.3390/brainsci13101364 (PMC10605482; doi:10.3390/brainsci13101364)
Supplement: Supplementary file 1 [file brainsci-13-01364-s001.zip › brainsci-2585309-supplementary.pdf]

Peripheral Blood and Cerebrospinal Fluid Levels of YKL-40 in Alzheimer's Disease: A Systematic Review and Meta-Analysis

Yuchen Zhang, Jinzhou Tian, Jingnian Ni, Mingqing Wei, Ting Li and Jing Shi *

Department of Neurology, Dongzhimen Hospital, Beijing University of Chinese Medicine, Beijing 100700, China; zhangyc@bucm.edu.cn (Y.Z.); jztian@hotmail.com (J.T.); jingnian_ni@hotmail.com (J.N.); mingqingwei001@126.com (M.W.); bjliting@yeah.net (T.L.)

* Correspondence: shijing87@hotmail.com

Supplementary Material

Search strategies

Search String in Pubmed:

(((((("Alzheimer Disease"[Mesh]) OR (Alzheimer disease[Title/Abstract])) OR (Alzheimer*[Title/Abstract])) OR (dementia[Title/Abstract])) OR (AD[Title/Abstract])) OR (cogniti*[Title/Abstract])) AND ((((((((((("Chitinase-3-Like Protein 1"[Mesh]) OR (Chitinase-3-Like Protein 1[Title/Abstract])) OR (Chitinase 3 Like Protein 1[Title/Abstract])) OR (CHI3L1[Title/Abstract])) OR (YKL-40[Title/Abstract])) OR (YLK 40[Title/Abstract])) OR (Cartilage Glycoprotein 39[Title/Abstract])) OR (Glycoprotein 39[Title/Abstract])) OR (GP-39[Title/Abstract])) OR (GP 39[Title/Abstract])) OR (CGP-39[Title/Abstract])) OR (CGP 39[Title/Abstract]))

Search String in Embase:

#1 'alzheimer disease'/exp

#2 'alzheimer disease':ab,ti OR alzheimer*:ab,ti OR dementia:ab,ti OR ad:ab,ti OR cogniti*:ab,ti

#3 #1 OR #2

#4 'chitinase 3 like protein 1'/exp

#5 'chitinase 3 like protein 1':ab,ti OR 'chitinase-3-like protein 1':ab,ti OR chi3l1:ab,ti OR 'ykl 40':ab,ti OR 'ylk 40':ab,ti OR 'cartilage glycoprotein 39':ab,ti OR 'glycoprotein 39':ab,ti OR 'gp 39':ab,ti OR 'cgp 39':ab,ti

#6 #4 OR #5

#7 #3 AND #6

Search String in Cochrane Library:

#1 MeSH descriptor: [Alzheimer Disease] explode all trees

#2 (Alzheimer disease OR Alzheimer* OR dementia OR AD OR cogniti*):ti,ab,kw

#3 #1 or #2

#4 MeSH descriptor: [Chitinase-3-Like Protein 1] explode all trees

#5 (Chitinase 3 Like Protein 1 OR CHI3L1 OR YKL-40 OR YLK 40 OR Cartilage Glycoprotein 39 OR Glycoprotein 39 OR GP-39 OR GP 39 OR CGP-39 OR CGP 39):ti,ab,kw

#6 #4 or #5

#7 #3 and #6

Search String in Web of Science:

#1 TS=(Alzheimer disease OR Alzheimer* OR dementia OR AD OR cogniti*)

#2 TS=(Chitinase-3-Like Protein 1 OR Chitinase 3 Like Protein 1 OR CHI3L1 OR YKL-40 OR YLK 40 OR Cartilage Glycoprotein 39 OR Glycoprotein 39 OR GP-39 OR GP 39 OR CGP-39 OR CGP 39)

#3 #1 AND #2

Table S1 Newcastle Ottawa Scale scores

ID	Author	Year	Population	Selection				Comparability		Exposure			Overall Quality
				1	2	3	4	5A	5B	6	7	8	

1	Lu	2019	China	*	*	*	*	*	*	*	*	8
2	Yasuno	2022	Japan	*	*	*	*	*	*	*	*	8
3	Villar-Piqué	2019	Germany	*	*	*	*		*	*	*	7
4	Ko	2021	Korea	*	*	*	*	*	*	*	*	8
5	Schulz	2021	Germany	*	*	*	*	*		*	*	7
6	Manniche	2020	Denmark	*	*	*	*	*		*	*	7
7	Toschi	2019	Sweden	*	*	*	*	*		*	*	7
8	Nordengen	2019	Norway	*	*	*	*	*	*	*	*	8
9	Morenas-Rodríguez	2019	Spain	*	*	*	*	*		*	*	7
10	Lleó	2019	Spain	*	*	*	*	*		*	*	7
11	Llorens	2017	Germany	*	*	*	*			*	*	6
12	Alcolea	2017	Spain	*	*	*	*	*		*	*	7
13	Janelidze	2016	Sweden	*	*	*	*	*	*	*	*	8
14	Olsson	2013	Sweden	*	*	*	*	*	*	*	*	8
15	Mattsson	2011	Sweden	*	*	*	*		*	*	*	7
16	Zhang	2018	USA	*	*	*	*	*	*	*	*	8
17	Kester	2015	Netherlands	*	*	*	*	*	*	*	*	8

1. Case definition is sufficient, with independent verification. 2. Continuous collection and representative cases. 3. Community control. 4. Control of disease history without neurological system. 5 A. Research controls MMSE. 5 B. Research controls other confounding factors. 6. Exposure is determined by reliable records. 7. The same method was used to determine the exposure of the case group and the control group. 8. None-response rates were similar for case and control groups. A study can be awarded a maximum of one star for each numbered item within the Selection and Exposure categories and a maximum of two stars for Comparability. Scores for low (0-3), moderate (4-6), and high-quality studies (7-9) were assigned.

Table S2 Sensitivity analysis of YKL-40 levels in peripheral blood

**Study omitted**	**Estimate**	**[95% Conf.**	**Interval]**
Lu 2019	0.49690172	0.28879547	0.70500797

Yasuno 2022	-0.22995767	-1.4604765	1.0005612
Schulz 2021	-0.31707555	-1.5550357	0.92088461
Villar-Piqué 2019	-0.33678982	-1.8411367	1.167557
Ko 2021	-0.34432453	-1.8594524	1.1708033
Combined	-0.16162802	-1.2023837	0.87912768

Effect sizes were pooled using random-effects mode

Table S3 Publication bias of YKL-40 levels in peripheral blood

A

Begg's Test

adj. Kendall's Score (P-Q)	=	-6
Std. Dev. of Score	=	4.08
Number of Studies	=	5
z	=	-1.47
Pr > |z|	=	0.142
z	=	1.22 (continuity corrected)
Pr > |z|	=	**0.221** (continuity corrected)

B

Egger's test

Std_Eff	Coef.	Std.Err	t	P>|t|	[95% Conf.Interval]	
slope	1.155721	1.318603	0.88	0.445	-3.040661	5.352103
bias	-4.900103	5.913361	-0.83	**0.468**	-23.71906	13.91885

Table S4 Sensitivity analysis of YKL-40 levels in CSF

**Study omitted**	**Estimate**	**[95% Conf.**	**Interval]**

Schulz 2021	0.88728368	0.64872688	1.1258404
Manniche 2020	0.92678702	0.69015503	1.1634191
Toschi 2019	0.88466585	0.64217782	1.1271539
Nordengen 2019	0.86188096	0.62421608	1.0995458
Morenas-Rodríguez 2019	0.8755728	0.62948966	1.1216558
Lleó 2019	0.84996116	0.61679399	1.0831283
Llorens 2017	0.88864118	0.6385228	1.1387595
Alcolea 2017	0.82446456	0.62263477	1.0262944
Janelidze 2016	0.90995312	0.66265684	1.1572495
Olsson 2013	0.93311441	0.70059526	1.1656336
Mattsson 2011	0.92675775	0.69341069	1.1601049
Zhang 2018	0.9214201	0.68533415	1.157506
Kester 2015	0.92082667	0.68036729	1.161286
Combined	0.89333507	0.66541034	1.1212598

Effect sizes were pooled using random-effects mode

Table S5 Publication bias of YKL-40 levels in CSF

A

Begg's Test

adj. Kendall's Score (P-Q)	=	-8
Std. Dev. of Score	=	16.39
Number of Studies	=	13
z	=	-0.49
Pr > |z|	=	0.625
z	=	0.43 (continuity corrected)
Pr > |z|	=	**0.669** (continuity corrected)

B

Egger's test

Std_Eff	Coef.	Std.Err	t	P>|t|	[95% Conf.Interval]	
slope	1.241916	0.4199218	2.96	0.013	0.3176742	2.166157
bias	-1.531967	1.978217	-0.77	**0.455**	-5.885993	2.822059

Table S6 Sensitivity analysis of overall YKL-40 levels

Study omitted	Estimate	[95% Conf.	Interval]

Lu 2019	0.79653746	0.59670353	0.99637133
Yasuno 2022	0.6309728	0.2856769	0.97626871
Schulz 2021 （Serum）	0.61344278	0.26576751	0.9611181
Villar-Piqué 2019	0.61240715	0.25409055	0.97072369
Ko 2021	0.61040348	0.24399297	0.97681391
Schulz 2021 （CSF）	0.58760452	0.2404515	0.93475747
Manniche 2020	0.61272949	0.25790489	0.96755409
Toschi 2019	0.58441877	0.23416188	0.93467563
Nordengen 2019	0.56631076	0.21893539	0.91368616
Morenas-Rodríguez 2019	0.57755798	0.22442576	0.93069023
Lleó 2019	0.56252104	0.21214518	0.91289687
Llorens 2017	0.5863651	0.22913665	0.94359356
Alcolea 2017	0.54357177	0.20887913	0.87826437
Janelidze 2016	0.59988403	0.23985121	0.95991689
Olsson 2013	0.61220974	0.25011507	0.97430438
Mattsson 2011	0.61883724	0.26940528	0.96826917
Zhang 2018	0.61387837	0.26359311	0.9641636
Kester 2015	0.60803425	0.25152871	0.96453977
Combined	0.60753191	0.27197884	0.94308499

Effect sizes were pooled using random-effects mode

Table S7 Publication bias of overall YKL-40 levels

A

Begg's Test		
adj. Kendall's Score (P-Q)	=	-15
Std.Dev.of Score	=	26.40
Number of Studies	=	18
z	=	-0.57
Pr > |z|	=	0.570
z	=	0.53 (continuity corrected)
Pr > |z|	=	**0.596** (continuity corrected)

B

Egger's test

Std_Eff	Coef.	Std.Err	t	P>|t|	[95% Conf.Interval]	
slope	1.351717	0.5364334	2.52	0.023	0.2145291	2.488905
bias	-3.052928	2.493958	-1.22	**0.239**	-8.339883	2.234027

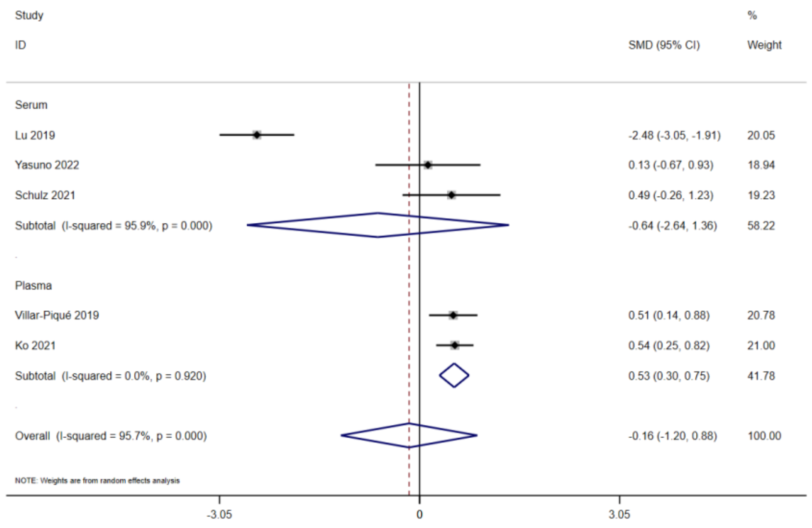

Figure S1. Forest plot of subgroup analysis of YKL-40 by sample in peripheral blood of AD patients and HCs.

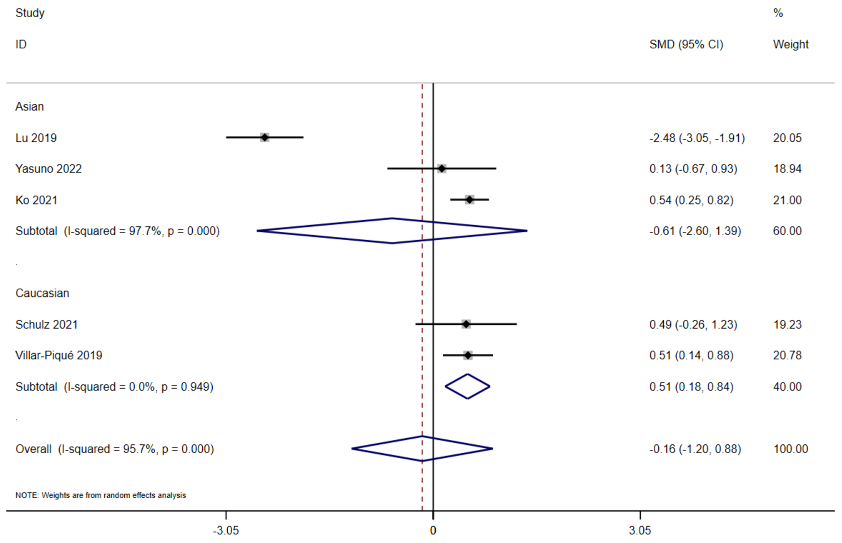

Figure S2. Forest plot of subgroup analysis of YKL-40 by ethnicity in peripheral blood of AD patients and HCs.

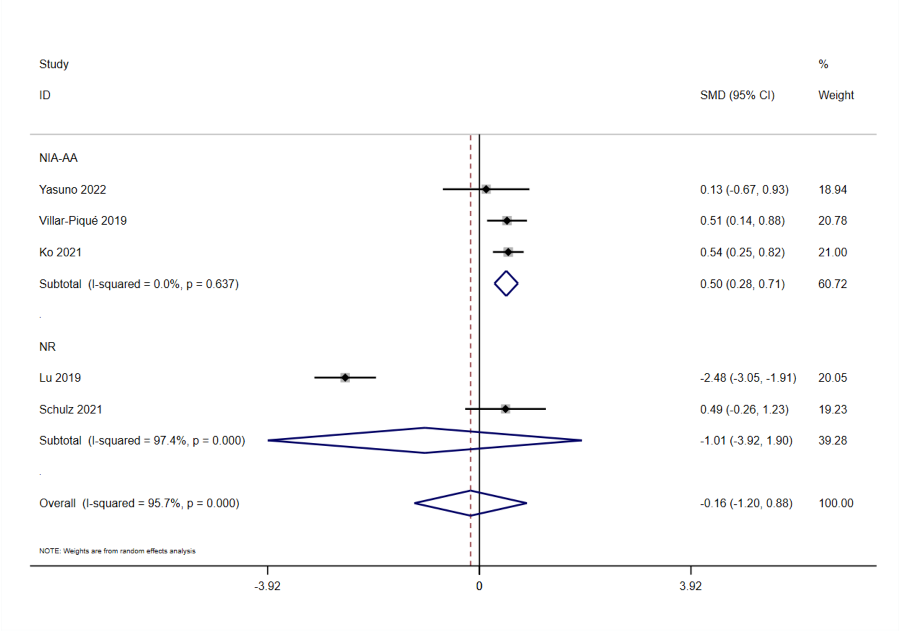

Figure S3. Forest plot of subgroup analysis of YKL-40 by AD criteria in peripheral blood of AD patients and HCs.

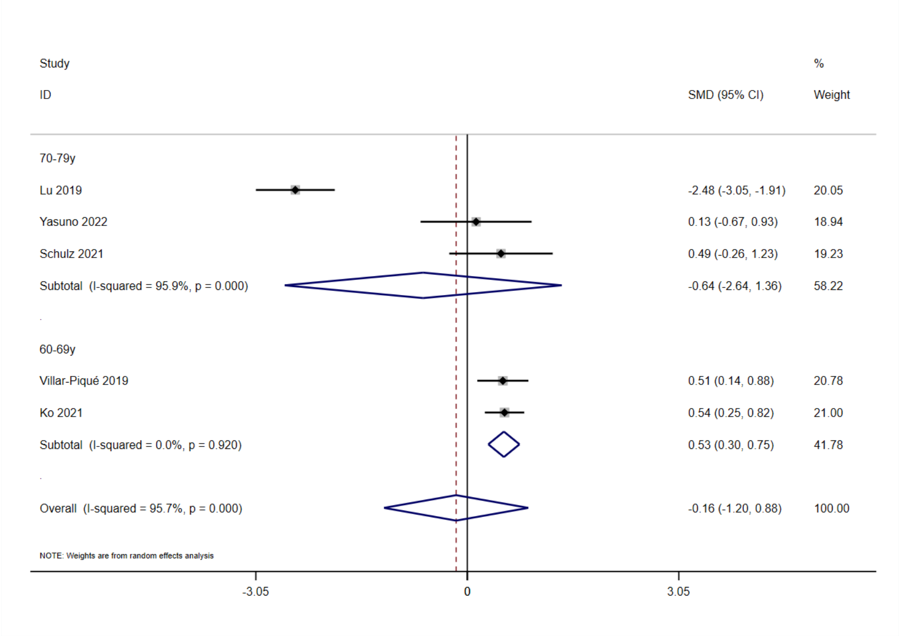

Figure S4. Forest plot of subgroup analysis of YKL-40 by mean age in peripheral blood of AD patients and HCs.

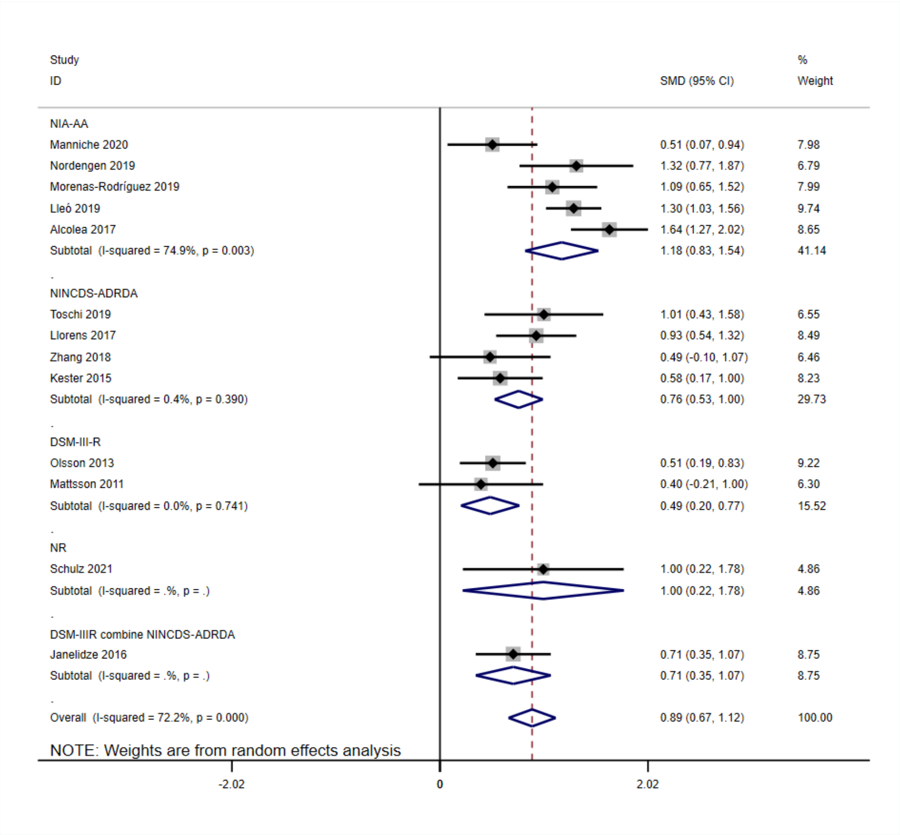

Figure S5. Forest plot of subgroup analysis of YKL-40 by AD criteria in CSF of AD patients and HCs.

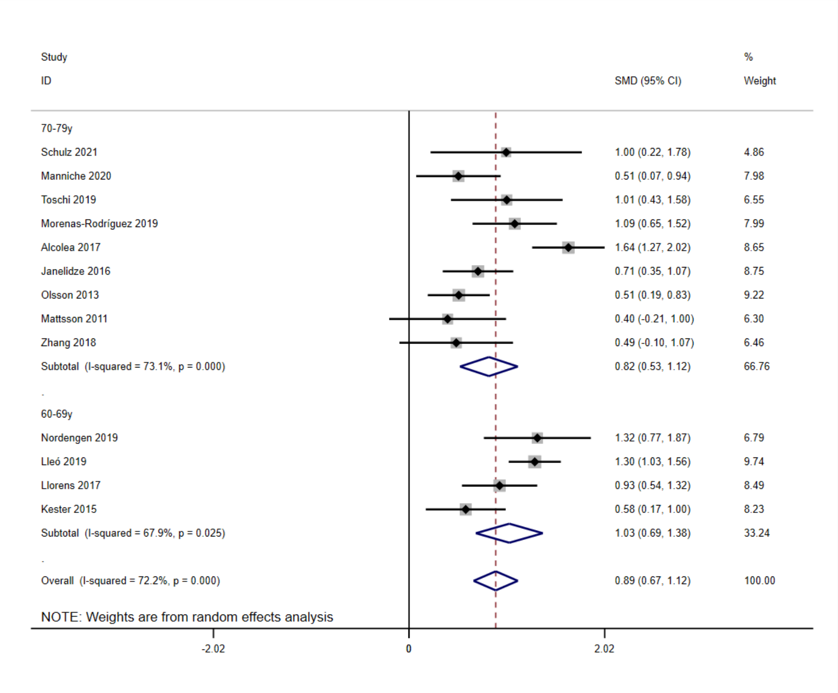

Figure S6. Forest plot of subgroup analysis of YKL-40 by mean age in CSF of AD patients and HCs.

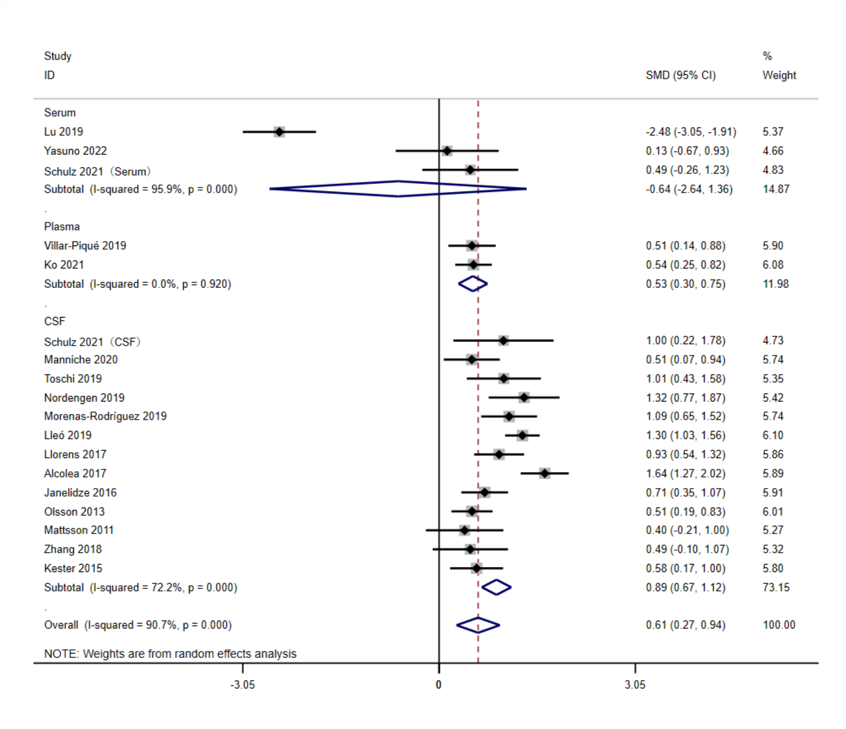

Figure S7. Forest plot of subgroup analysis of overall YKL-40 by sample of AD patients and HCs.

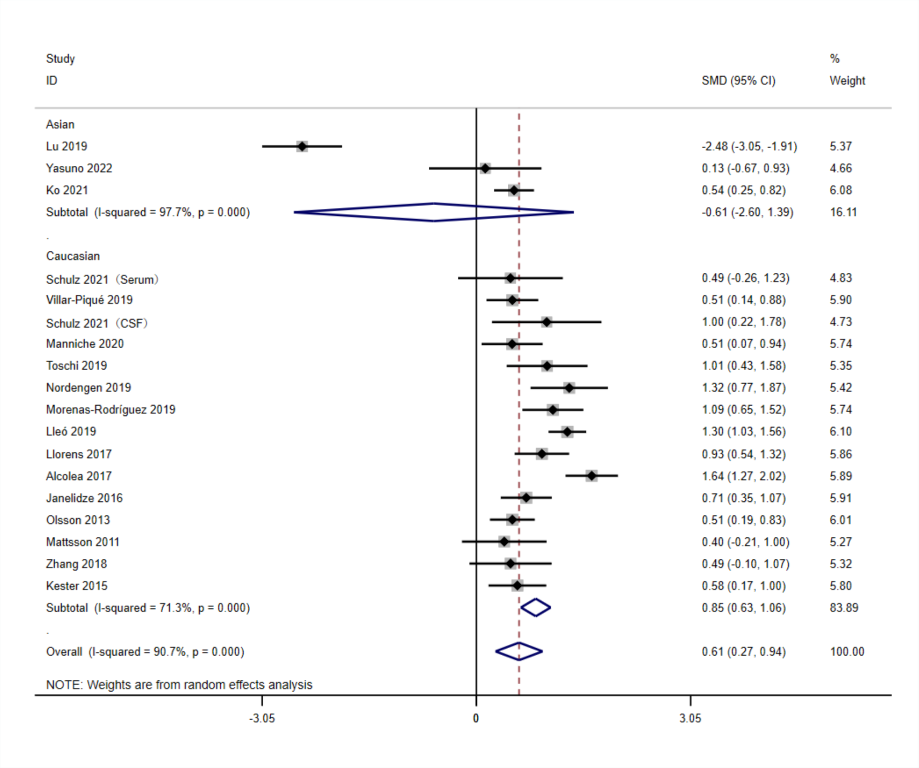

Figure S8. Forest plot of subgroup analysis of overall YKL-40 by ethnicity of AD patients and HCs.

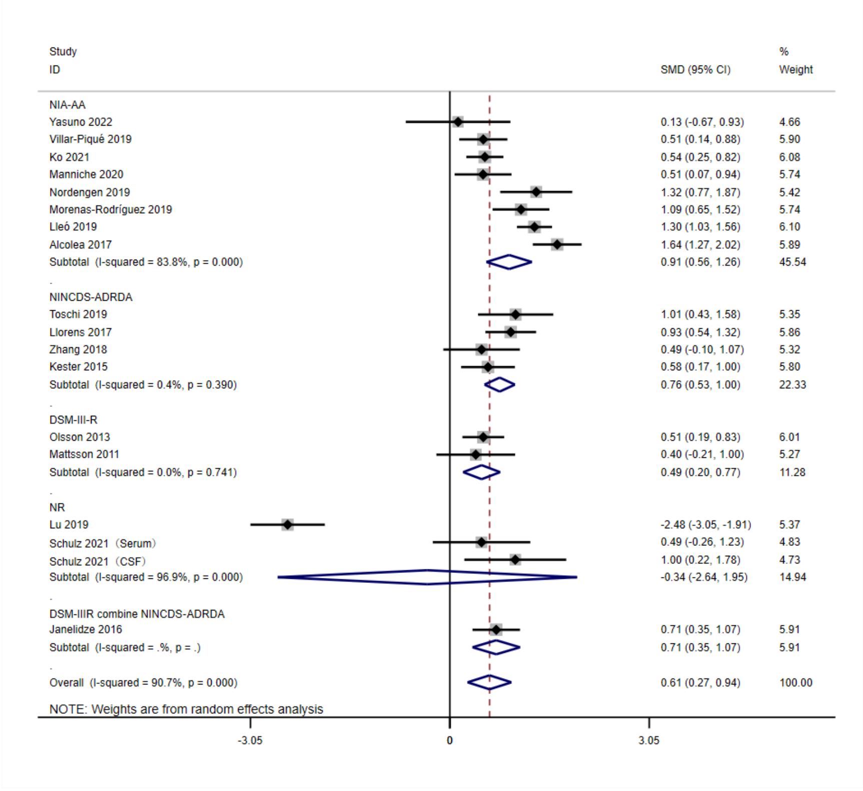

Figure S9. Forest plot of subgroup analysis of overall YKL-40 by AD criteria of AD patients and HCs.

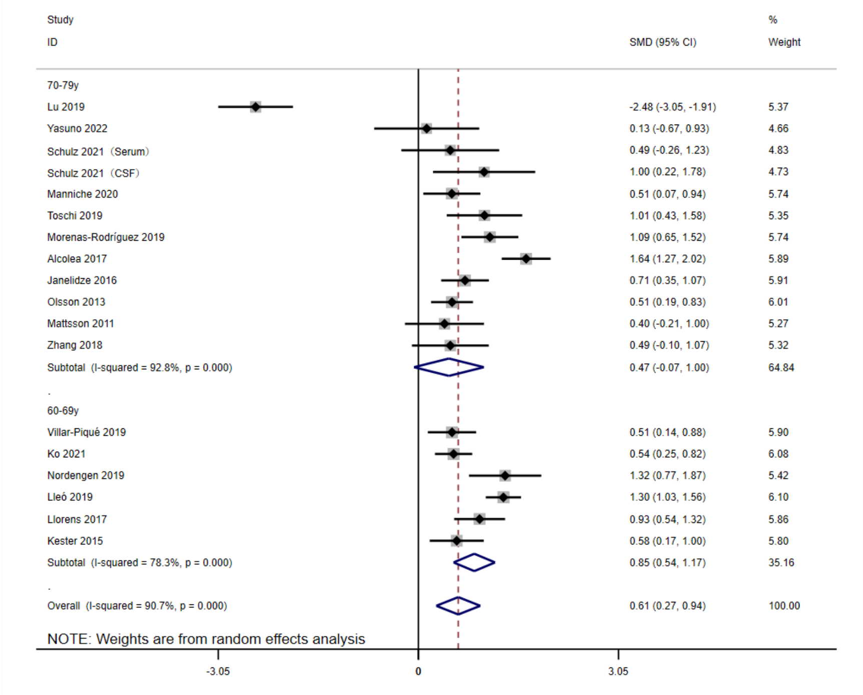

Figure S10. Forest plot of subgroup analysis of overall YKL-40 by mean age of AD patients and HCs.
